# Researches on the Development of Tuberculous Matter

**Published:** 1862-03

**Authors:** 

**Affiliations:** Professor in Val-de-Grace


### RESEARCHES ON THE DEVELOPMENT OF TUBERCULOUS MATTER.

By DR. LAVARAU,

*Professor in Val-de-Grace.*

Microscopical studies have followed, in France and Germany, two different paths. Less prompt in making use of this marvelous means of analysis, we have applied it with a sort of distrust which has kept us in the circle of precise determinations; in Germany the same success of microscopy seems to have drawn many into the error of attributing to theoretic explanations, founded on a progressive science, the value of the facts which only owe their importance to the method which has produced them. For our neighbors, the cell is the point of departure for all organic processes, the centre of a local irritability and a circulation more wonderful than that of the absorbent and exhalent vessels of Bichat,—and in fine a being in a being, since the cell partakes with the egg and grain of seed the characteristic of being produced always from a pre-exisiting cell. *Omnis cellula a cellula* is henceforth the formula of a new medical doctrine which excludes all interpretation borrowed from the doctrine of exhalation and hyperæmia. Like everything which emanates from its author, the cellular doctrine is as remarkable for the ingenuity of the views as for the truth of the observation. It furnishes numerous and important facts, gives a more complete knowledge of the inflammatory process, and the relations of pathological formations with normal nutrition; but it seems to us to serve in suppressing from physiology hyperæmia and hypercrinia. How can we explain the fibrinous formations on the mucous and serous membranes without a physiological interpretation as well founded as that which invokes conditions of irritation in the cells themselves? How are we, in taking no account of it in tuberculization, or rather in passing by a doctrine so important without examination, or adopting it without submitting it to a sufficient criticism? I have, therefore, thought that the subject of tubercle one of the anatomo-pathological questions the most worthy of interest, would not be uninteresting in presence of the cellular doctrine.

*Opinion of Authors on Development of Tubercle.*—The opinions on the mode of development of tubercle may be reported in two different points of view. For one party, tubercle is the product of a morbid exudation; for the other, the consequence of atrophic degeneration of the normal elements of our tissues. The first opinion is generally that of the French physicians, and of German physicians of the Vienna school, and of some English physicians, as Bennett, Ancell. MM. Andral, Lebert and Mandl consider tubercle as a product of secretion. "After its excretion, tubercle takes at first a compact form," says Lebert. "We may affirm," says Mandl, "the tuberculous matter was primarily liquid; coagulation takes place immediately after exudation." According to Gerber, fibrinous tubercle comes from exuded plastic matter which has not been reabsorbed nor transformed into pus. "With regard to the origin of tubercle," says Vogel, "we can not doubt that the substance which produces it may be furnished in the liquid state by the capillary vessels." Albers and Czermack hold the same opinion. Rokitansky considers tubercle as produced by the exudation of a particular nature.

*Metamorphosis.*—The opinion which attributes the development of tubercle to the atrophic metamorphosis of the elements of our tissues has been introduced into our science by Henle in Germany, and Addison in England. Henle applies to tubercle the opinion emitted by Muller on cancer; viz: that the formations are composed of primary cells, more or less altered. Addison attributes the production of tubercle to the metamorphosis of the white globules of the blood. When the normal elements undergo their metamorphosis in an incomplete manner, the normal products are replaced by those of a retrograde nature. M. Kuss, of Strasburgh, has published in France the ideas of the German school. He expresses his opinion in the following language:

"In the beginning, the epithelial globule preserves its principal optic properties—it remains transparent; from this the initial forms of grey granulation, of infiltrated gelatinous tubercle. Later, the slow accumulation compresses, wears out and causes the skeleton of the lungs to disappear; that is to say, the vascular membrane of the cells, then the epithelial globule, after a certain lapse of time, dies, mummifies, shrivels, changes its optic properties, and becomes more opaque. It is this form which has been taken for the corpuscles of tubercle. As to tubercle of other organs, it is also the result of mummification of normal tissues under the form of elementary globules. We may then define tuberculization, the death and the mummification of a normal tissue, or accidentally characterized by small globules like to those of the pulmonary parenchyma of the kind that Henle calls *elementary corpuscles.*"

For Reinhardt, tubercle is the product of fatty degeneration of epithelial cells. Shroeder Von der Kolk considers yellow tubercle the result of the swelling of the epithelial cells by plastic matter. Finally Virchow attaches to this opinion the authority of his name in different publications, especially in his *Cellular Anatomy*, on page 399. In my opinion, says Virchow, tubercle is a granule, or a knot, and this knot constitutes a new formation, which at the moment of its first development possesses necessarily the cellular structure, and springs, like the other new formations, from the connective tissue. When this new formation has arrived at a certain stage of its development, it shows, in the middle of the normal tissue which it occupies, a small prominent knot composed of small cells with one or several nuclei. That which characterizes especially the new formation is its richness in nuclei, and when we observe it in the surface of the tissue we see almost nothing but nuclei. If we isolate these products, we find either small elements with nuclei so small that the membrane lies directly in contact with the nuclei, or more voluminous cells, in which the nuclei are divided, and may be seen to the number of twelve, twenty-four, or thirty, in a single cell; the nuclei are small, homogeneous, and of an aspect but slightly shining. This stricture, which in its development is comparatively most nearly related to pus, inasmuch as it has the smallest nuclei and relatively the smallest cells, is distinguished from all the more highly organized form of cancer, cancroid and sarcoma, by the circumstance that these contain large, voluminous—nay, often gigantic—corpuscles with highly developed nuclei. Tubercle, on the contrary, is always a pitiful production, a new formation from its very outset miserable.

The difference of opinions on the question of pathologico-physiology relative to the development of tubercle is equally exhibited on the question of its nature and appearance.

*Organized Product.*—For one party, tubercle is organized, containing cells with nuclei. Geber distinguishes non-organized albuminous tubercles from fibrinous tubercles constituted by tubercles containing cells with a nuclei having the power to organize themselves into fibres. Gellerstedt considers tubercle as living the same life as tissues deprived of capillaries, as the nails and hair.

For Rokitansky, tubercle forms the transition of non-organized to the organized productions. Schroder Von der Kolk believes, that grey tuburcle may transform itself into cellular fibres.

*Non-Organized Product.*—The largest number of microscoscopists regard tubercle as a product of excretion, presenting no one of the attributes of organization. For Koestlin, tubercle never raises itself beyond an inferior degree of organization of an amorphous substance containing nuclear elements. This is also the opinion of John Simon, Henle, Kuss and Virchow, who regards it as the product of decomposition of normal cells; and especially of M. Mandl, who sums up his opinion on its pathological anatomy in the following language:

"The tuberculous matter is not composed of elements which may increase and develope themselves. The multiplications and increase of tubercles can not consequently be explained except by juxtaposition. Tuberculous matter is a non-organized amorphous matter."

*Aspect of Tuberculous Matter.*—For one party, tubercle is characterized by a special histological element; for the other, by the amorphous matter. In the first party are found Kuhn, Gluge, and especially M. Lebert, who attributes to tubercle—1st, elementary granules; 2nd, a solid amorphous blastema; 3d, characteristic cells. In crude tubercle the tuberculous globule offers irregular outlines, approaching either a spherical or an oval form; it is ordinarily irregular, angular, polyhedric, with rounded angles and corners of a diameter of 0·005 to 0·0075, approaching rarely to 0·01. According to Paget, Madden admit the reality of charactericteristic globules—Bennett only giving a diameter to the tuberculous globules of 0·01. According to Paget, it is formed of fatty granules, of nuclei of different aspects, and finally by veritable cells, which are only epithelial cells transformed into tuberculous cells.

*Amorphous Substance.*—Koestlin, of Stuttgard, Rokitansky, and M. Mandl admit that the tuberculous substance is contributed by an amorphous blastema, containing or not containing altered cells. Tubercle, according to Virchow, has no characteristic element, properly so called; the atrophied nuclei which result from the decomposition of the cells are the only elements which maintain their characteristics. According to M. Mandel, "the tuberculous substance is an amorphous mass studded with fatty molecules, finely granulated, cohering at the beginning of its existence, defluent later." The fragments of this substance presents neither determined forms nor sizes. Particular tubercular globules or corpuscules have no existence.

In the face of views and opinions so different and opposite, I shall try to give my own opinion on this, one of the most important questions in pathological anatomy.

*Personal Observations and Reflections.*—Tubercle in its first stage of development is constituted by a grey homogeneous substance, whitish, elastic, developed either on some isolated point of the organism, oftenest producing itself, like the disseminative inflammations (dothin-enteritis, variola), on a great number of points at once, without being characteristic of tubercle, as of cancer and pus; that this dissemination of the pathological product may appear consecutive to a secondary process of reabsorption or to any other mode or reproduction, the simultaneous evolution of the tuberculization being evidence neither of an alteration of the blood nor of the nutritive fluid.

Tubercle does not develope itself in all the tissues nor in all the organs. Differing from pus and cancer, it never commences in the epithelial cells; it does not develope itself in the muscular tissue, nor perhaps in the nervous tissue, nor in points of the consecutive tissue, where the fibres form compact fascicula, as in the tendons, the aponeuroses, and the skin—however, these are precisely the parts of this tissue where the plasmatic cells take their greatest development. Generally we observe it in the organs where there exists a vascular network with thin sides, in connection with physiological exudation, with gaseous endosmose where from easy communication, as in the lungs, the lymphatic vessels, the follicles, and the plates of Peyer, veritable lymphatic glands flattened according to Brucke, who has observed the same globules in in their interior, as in the ganglions, the serene membranes, and finally in the kidneys and testicles.

Tubercle is rare in the liver, which, perhaps on account of the capsule of Glisson, partakes with the most part of the grape-like glands the character of being rarely attacked with this kind of lesion, without our being able to attribute to something else than the predominance of fibrous element the absence of a product which develops itself so frequently in the glands of the large intestines and the glands of Lierberkiehn, so like by the general disposition of the grape-like glands to the greatest fibrous development, as the salivary and mammary glands. In this point of view we very easily perceive a certain relation between tuberculization and the existence of a system of vessels abundantly disposed, as to their walls, for a destination either of gaseous endosmose or secretory excretion. Bennett, in England, has been struck with this relation; he considers tubercle as a product of exhalation in the parts where the vessels present the least consistence. On the other hand, Baron, in England, M. Cruveilhier and Becquerel, in France, have insisted on the relations of adhesion and proximity which exist between tubercle and the venous vascular system; relations perceptible to the eye on the meninges, since it is universally admitted at present that the tuberculous granulations of the pia-mater have their seat in the inferior cerebral veins, anterior and median, and the cerebellar; and that we may observe equally through a magnifying glass on a layer of the spleen and the cortical tissue of the kidney, which becomes very apparent when there exists at the same time pulmonary granulations and an œdema of the lungs; we may then by scraping raise veinous portions which sustain grape-like clusters of granulations manifestly adherent. Blainville regarded fat as furnished by the black blood, and exhaled through the sides of the veins. He was led to this opinion by the attentive observation of the manner in which fat is distributed in the epiploon. It is difficult not to remember the opinion of the ingenious physiologist in studying the relations with the veins of tubercles, which contain so much fatty matter. Moreover, the impermeability of tubercles to fine injections, tried in vain by MM. Natalis-Guillot and William Starck; the frequency of hæmorrhages and dropsies determined by this lesion, confirm the existence of certain relations between tubercle and the vascular system.

Let us see what the microscope reveals. The microscopists have advanced different opinions on the anatomical element which serves for the support of the tuberculous substance. According to Lebert, (*Anatomie Pathologie*,) tubercle developes itself sometimes in the intervesicular tissue, sometimes in the vesicles themselves. According to Schroeder Von der Kolk, grey tubercle is seated in the interstitial tissue of the lungs, and the yellow in the interior of the vesicles. MM. Kuss and Reinhardt place their seat in the interior of the cells. Virchow admits that tubercle developes itself equally in pathological and normal tissues, in the transitory cellular parts as well as the permanent fibrous organs. Robert Carswell holds that the free surfaces of the mucous membranes serve as the principal seat for the development of tubercle; he comprises under the name of free surfaces the bottom of the cul-de-sac of tubular glands. Finally, Mandl regards the tuberculous plasma as having the power to infiltrate itself between all the elements of the pulmonary tissue and penetrate into their intimate structure.

For more than ten years that I have examined all the aspects of the question of tuberculization, I have found no fact which permits me to doubt that tubercle may have its seat elsewhere than in the interstitial tissue of the lungs. If tubercle developes itself either in the interior of the vesicles or in the thickness of the epithelial cells, we find it in the thickness of the granulations, or in the fasciculi of the elastic fibres which limit the pulmonary cells or the epithelial cells. Now I affirm it is never to be found thus; besides, if we transfer the question on the ground of the serous membranes which the fibrous tissue, no more plated in lobules but spread out in a membrane, the question produces itself with a solution in the most evident manner.

*Preparation.*—We detach with care a portion of the serous membrane covered with tuberculous granulations as slightly developed as possible, and after having extended it by the aid of pins on a small piece of cork, we submit it to dessication for some hours, taking care to mark by two lines crossing each other, traced with a fine pencil, the precise point of the granulation which, by the drying, confounds itself to the naked eye and magnifying-glass with the surrounding parts. We detach with a very sharp instrument some small bits from both sides, and it is then easy to observe that on the free side the epithelial cells present themselves with their volume and their normal transparency; whilst in carrying under the eye-piece the deeper bits we perceive by the side of the transparent spaces traversed by vessels, spaces whose opaline appearance proves the presence of tuberculous matter. These are not, moreover, ever penetrated by the vessels, which stop at their limit. It is impossible to doubt, after the preceding preparation, that tuberculous matter does not deposit itself on the extremity of the vascular branches in the simple connective tissue (tissue of Reichert), or between the connective fibrilli.

*Aspect of Grey Tubercle.*—Under a magnifying power of three hundred diameters, a small bit, slightly moistened, covered with an object-glass, and drawn slowly through the field of the eye-glass, offers transparent spaces, with folds simulating fibres, (connective tissue of Reichert,) very distinct fibres, on the track of which appear at distant intervals the cells recognized by Donders, and to which Weber (of Bonn) and Virchow have attached so much importance, and opaline parts corresponding to the presence of tuberculous matter. In lengthening and shortening the focus by gentle movements of the screw, we distinguish, through an opaline granulous blastema, appearances of badly-formed cells, badly circumscribed, of $0^{mm}$, 002 to $0^{mm}$, 003 of a milimetre, which I compare to similar dispositions which simulate cells in the homogeneous tissue of the acephalocysts, or in the sarcode of the infusoria.

If we submit the preparation for some time to the action of pure acetic acid, the opaline parts become transparent, presenting superposed lines, simulating fibre; the cells disappear, and drops of fat can be seen swimming around the preparation, varying from $0^{mm}$, 04 to $0^{mm}$, 02 in diameter. The preparation resembles the aspect of albumino-fibrinous productions, less the leucocysts, and with more fatty drops. The tuberculous drops described by M. Lebert appear to me to belong to a more advanced degree of development, or of degeneration of the tuberculous matter; very charactistic, they appear as small round or oval fragments, with a nucleus, with an elevated contour, affecting an aspect which resembles somewhat the cartilaginous cells. In the tubercle, in process of softening, we find the same globules more isolated on the border of a granulated amorphous mass, containing in its thickness the same globules which we perceive by transparence in moving the eye-piece by the screw. In giving a movement to the parts submitted to observation, we see the globules fly into other fragments, smaller or more voluminous, in such a way as to resemble very much the breaking up of the ice on the surface of a river where the movement of fragments of altered fibrine borrowed from a hematic tumor in process of decomposition takes place.

When the softening is complete, there exists in the middle of the tuberculous fragments leucocysts proceeding, as well as fragments of fibres, from the surrounding tissues.

If from the study of the lesion we seek to advance to the notion of pathological physiology, which can only end in a medical idea, it is indispensable that, conforming to the rules of the method, we should embrace at once all the characters particuler to the tubercle, in establishing what is proper to it, and what distinguishes it from other pathological products. Tubercle differs from pus, because pus has for principal element the leucocyst, which belongs to the normal life of blood; that pus, the product of a process of prolifiration, which we are able to provoke, developes itself in all the tissues and in all the organs. It differs from cancer, because cancer is organized, that it effects especially the epithelial cells, the grapiform glands, and the fibrous element of the connective tissue.

Cancer appears to attach itself in its development to a state of organic decay (old age). Tubercle seems rather connected with a state of organic evolution. Developed in the organs where there exists an abundant vascular network, it affects in its evolution a great many distinct points, realizing the pathological idea of the diathesis. Cancer, on the contrary, at first local, generalizes itself secondarily by a process of resorption or reproduction which resembles rather the idea of cachexia. Cancer, in so much as an organized product, carries in itself the fatal conditions of its increase and of its extension; simple tubercle, a fereign body, may remain without action in the middle of our organs, on which it does not act, moreover, but as an agent of compression or irritation. Cancer does not undergo any other influence in its march and its development than that of individual conditions. Tubercle, although appealable to predispositions of race and family, is submitted in its progress, and probably its evolution, to external modifying causes. We know how fatal the catarrhal constitutions are to tuberculous persons, and we have personally seen how much the conditions which provoke scurvy react fatally on the same patients. Laennec and M. Louis have then justly raised to the rank of disease a lesion, perfectly characterized by its mode of development, its physical aspect, and its progress, which ends either in a calcareous transformation, or in a fatty degeneration, with softening.

If it was permitted us, in our day, not to be carried away in the precise terms of a definition, and allow our thoughts to wander beyond the limits where the knowledge of the times retains us, we would say that tubercle seems to us to be produced by a state of nutritive fluid, which resembles the degree of degradation this fluid undergoes when, being no more represented by the blood, the lymph, and the serosity, which are the three aspects under which it produces itself in the superior animals, it reaches a state of being no longer constituted than by a sarcodic humor containing some plasmic cells. Finally, in every point where tubercle develops itself, the circulation is arrested, irritability is extinguished. Instead of the process of physiological activity and renovation of the parts, an inert mass constitutes itself, and absorbs the normal parts; instead of the interior movement of vital expansion, the normal product increases itself by juxtaposition, to that point, that, acting on the surrounding parts, it becomes for it a cause of destruction.—*Gaz. Hebdomadaire, Sept. 20th.—Translated by Cincinnati Lancet & Observer.*

